# Triage of patients for emergency medical team based on pre-hospital observations

**DOI:** 10.1186/1757-7241-21-S2-A50

**Published:** 2013-09-09

**Authors:** Christian Melchior Olesen, Christian Baaner Skjærbæk, Leif Rognås

**Affiliations:** 1 Department of Anesthesiology, Viborg Hospital, Denmark; 2 Department of Medicine, Viborg Hospital, Denmark

## Background

Critically ill patients are likely to benefit from being received by a predefined multidisciplinary team providing a structured and well qualified initial examination and immediate treatment of life-threatening conditions.

At Viborg Hospital all critically ill non-trauma adult patients have been received by an emergency medical team (EMT) since January 1st 2012. The EMT is a preformed multidisciplinary team led by senior registrars or consultants from the Department of internal medicine and the Department of Anesthesiology and Intensive Care. To identify those patients that should be received by EMT and ensure optimal resource utilization a triage system is necessary. Based on the triage system ADAPT, we have developed a system where data from the Emergency Medical Services form the basis for the triage.

To our knowledge, no other Danish study have evaluated the use of pre-hospital data for the triage of unselected critically ill non-trauma patients.

We aimed at evaluating the present triage model.

## Methods

In all EMT activations we recorded the patients’ vital status, the preliminary diagnosis and the patients’ transfer destinations when leaving the emergency department. The physicians assessed the relevance of team activation.

## Results

269 AMT-activations were recorded. The activation was classified as relevant in 248 cases (92%). 141 patients (52%) were transferred to the intensive care unit. 94 patients (35%) were transferred to the emergency medical ward or the cardiology department. 8 patients (3%) were transferred to the surgical room and 7 patients (3%) were transferred directly to another hospital.

## Conclusion

We found that the triage-model effectively identified patients for whom EMT-activation would not be relevant. This conclusion is supported by the fact that a large proportion of the patients treated by the EMT needed intensive care.

The study weakness is that we have no assessment of undertriage.

In an attempt to estimate this possible undertriage, we are currently conducting a study of non-trauma patients who died or were transferred to the intensive care unit within the first 24 hours after admission to identify whether they were treated by the EMT or not.

